# Complete genome sequence of *Bacillus pumilus* X14-67 with antimicrobial properties isolated from aged *Pericarpium citri reticulatae* “Chachiensis”

**DOI:** 10.1128/mra.01151-24

**Published:** 2025-01-07

**Authors:** Qianxian He, Shijie Xiong, Gu Chen

**Affiliations:** 1 School of Food Sciences and Engineering, South China University of Technology, Guangzhou, China; Portland State University, Portland, Oregon, USA

**Keywords:** *Bacillus pumilus*, *Pericarpium citri reticulatae*, bacteriocin, genome sequence

## Abstract

*Bacillus pumilus* X14-67 was isolated from *Pericarpium citri reticulatae* “Chachiensis,” the natural aged pericarps of *Citrus reticulata* Blanco. It had inhibitory activity against *Staphylococcus aureus*, *Escherichia coli*, *Salmonella paratyphi*, and *Candida albicans*. Here, its genome was sequenced and annotated to understand its antimicrobial properties.

## ANNOUNCEMENT

*Pericarpium citri reticulatae* (PCR, in Chinese Chen Pi) is the dried and aged pericarps of *Citrus reticulata* Blanco and its cultivars, while PCR-Chachiensis (in Chinese Guang Chen Pi) is the most premium one. They have been used as food additives and medicine to cure gastrointestinal diseases such as stomachache, abdominal distension, and indigestion for hundreds of years (1). Our previous study revealed the central bacterial community and identified probiotic bacterial strains in PCR-Chachiensis (2, 3). As a natural aging product, PCR-Chachiensis might contain natural isolate with antimicrobial activity to maintain its central microbe community during aging and contribute to its premium quality.

*Bacillus pumilus* X14-67 was isolated from PCR-Chachiensis collected from orchard of Xinhui District in Guangdong Province of China on LB (lysogeny broth) and identified as *B. pumilus* with 16S rRNA gene sequencing (3). It had inhibitory activity against *Staphylococcus aureus*, *Escherichia coli*, *Salmonella paratyphi*, and *Candida albicans* obtained from Guangdong Microbial Culture Collection Center. A spot-on-lawn assay was used by spreading each test strain onto LB agar plate followed by spotting X14-67 in disc diffusion in the center. After incubation at 30°C for appropriate time, the clear inhibition zone between X14-67 and each test strain was measured. X14-67 had inhibition zone as large as 26.3 ± 2.3, 12.5 ± 2.2, 13.3 ± 1.3, and 16.1 ± 2.1 mm against *Staphylococcus aureus*, *Escherichia coli*, *Salmonella paratyphi*, and *Candida albicans*, respectively.

X14-67 genomic DNA was prepared from an overnight LB culture using STE method (4) and quantified by Nanodrop2500. Whole-genome sequencing was performed by combining PacBio with Illumina sequencing at Majorbio Bio-pharm Technology co. LTD (Shanghai, China). Genomic DNA was segmented into 8–10 kb using the G-tubes (Covaris, MA) method and following annealing to construct SMRTbell library using the SMRTbell Express Template Prep Kit (PacBio, CA) for PacBio single-molecule sequencing. For Illumina sequencing, genomic DNA was fragmented by sonication to construct a library using the NEXTflex Rapid DNA-Seq Kit (Revvity, MA) and then amplified by bridge PCR and sequenced by Illumina. Raw reads were trimmed and filtered with Filtlong v0.2.1. Default parameters were used for all software unless otherwise specified. A total of 7,015,534 and 616,295 clean reads, as well as 1,072,161,970 and 5,651,361,659 clean bases, were obtained from Illumina and PacBio sequencing, respectively.

SOAPdenovo (5) was used to assemble genome as one chromosome of 3,714,404 bp, with a sequencing depth of 1521.47 and 41.82% GC content. A total of 3,654 protein-coding sequences, 24 rRNA genes, and 81 tRNA genes were annotated using National Center for Biotechnology Information (NCBI) Prokaryotic Genome Annotation Pipeline v6.8 (6). Several promising biosynthetic gene clusters (BGCs) for secondary metabolite were identified by AntiSMASH (7), such as those similar to the BGC for lichenysin and bacillibactin. BAGEL4 (8) further identified three bacteriocin-encoding gene clusters, including those similar with pumilarin and amylocyclicin circular bacteriocin coding gene, respectively. They might contribute together to the antimicrobial properties of X14-67.

The antimicrobial properties and genomic information of X14-67 suggest its potential application in foodborne pathogens control and provide a new insight to study the beneficial characteristics of PCR-Chachiensis.

## Data Availability

The complete genome sequence of *Bacillus pumilus* X14-67 was deposited in GenBank (accession number: CP170098). The associated BioProject and BioSample accession numbers are PRJNA1162269 and SAMN43805711, respectively. Raw sequence reads have been submitted to the Sequence Read Archive (accession number: SRX26693893).
